# Risk of Early Versus Later Rebleeding From Dural Arteriovenous Fistulas With Cortical Venous Drainage

**DOI:** 10.1161/STROKEAHA.121.036450

**Published:** 2022-04-14

**Authors:** Andrew J. Durnford, Danyal Akarca, David Culliford, John Millar, Ridhima Guniganti, Enrico Giordan, Waleed Brinjikji, Ching-Jen Chen, Isaac Josh Abecassis, Michael Levitt, Adam J. Polifka, Colin P. Derdeyn, Edgar A. Samaniego, Amanda Kwasnicki, Ali Alaraj, Adriaan R.E. Potgieser, Stephanie Chen, Yoshiteru Tada, Ryan Phelps, Adib Abla, Junichiro Satomi, Robert M. Starke, J. Marc C. van Dijk, Sepideh Amin-Hanjani, Minako Hayakawa, Bradley Gross, W. Christopher Fox, Louis Kim, Jason Sheehan, Giuseppe Lanzino, Akash P. Kansagra, Rose Du, Rosalind Lai, Gregory J. Zipfel, Diederik O. Bulters

**Affiliations:** Wessex Neurological Center (A.J.D., D.A., J.M.), University Hospital Southampton, United Kingdom.; University of Southampton (D.C.), University Hospital Southampton, United Kingdom.; MRC Cognition and Brain Sciences Unit, University of Cambridge, United Kingdom (D.A.).; Department of Neurological Surgery, Washington University, St. Louis, MO (R.G., G.J.Z.).; Department of Neurological Surgery (E.G., W.B., G.L.), Mayo Clinic, Rochester, MN.; Department of Radiology (E.G., W.B., G.L.), Mayo Clinic, Rochester, MN.; Department of Neurological Surgery, University of Virginia, Charlottesville (C.-J.C., J.S.).; Department of Neurological Surgery (I.J.A., M.L., L.K.), University of Washington, Seattle.; Stroke and Applied Neuroscience Center (M.L., L.K.), University of Washington, Seattle.; Department of Neurological Surgery, University of Florida, Gainesville (A.J.P., W.C.F.).; Department of Neurology (C.P.D., E.A.S., M.H.), University of Iowa, Iowa City.; Department of Radiology (C.P.D., E.A.S., M.H.), University of Iowa, Iowa City.; Department of Neurological Surgery, University of Illinois at Chicago (A.K., A.A., S.A.-H.).; Department of Neurological Surgery, University Medical Center Groningen, Netherlands (A.R.E.P., J.M.C.v.D.).; Department of Neurological Surgery, University of Miami, FL (S.C., R.M.S.).; Department of Neurosurgery, Institute of Biomedical Biosciences, Tokushima University Graduate School, Japan (Y.T., J.S.).; Weill Institute for Neurosciences, Department of Neurosurgery, University of California San Francisco (R.P.).; Department of Neurological Surgery, University of Pittsburgh, PA (A.A., B.G.).; Mallinckrodt Institute of Radiology, Washington University, St. Louis, MO (A.P.K.).; Department of Neurosurgery, Brigham and Women’s Hospital, Boston, MA (R.D., R.L.).

**Keywords:** drainage, fistula, hemorrhage, incidence, natural history

## Abstract

**Background:**

Cranial dural arteriovenous fistulas with cortical venous drainage are rare lesions that can present with hemorrhage. A high rate of rebleeding in the early period following hemorrhage has been reported, but published long-term rates are much lower. No study has examined how risk of rebleeding changes over time. Our objective was to quantify the relative incidence of rebleeding in the early and later periods following hemorrhage.

**Methods:**

Patients with dural arteriovenous fistula and cortical venous drainage presenting with hemorrhage were identified from the multinational CONDOR (Consortium for Dural Fistula Outcomes Research) database. Natural history follow-up was defined as time from hemorrhage to first treatment, rebleed, or last follow-up. Rebleeding in the first 2 weeks and first year were compared using incidence rate ratio and difference.

**Results:**

Of 1077 patients, 250 met the inclusion criteria and had 95 cumulative person-years natural history follow-up. The overall annualized rebleed rate was 7.3% (95% CI, 3.2–14.5). The incidence rate of rebleeding in the first 2 weeks was 0.0011 per person-day; an early rebleed risk of 1.6% in the first 14 days (95% CI, 0.3–5.1). For the remainder of the first year, the incidence rate was 0.00015 per person-day; a rebleed rate of 5.3% (CI, 1.7–12.4) over 1 year. The incidence rate ratio was 7.3 (95% CI, 1.4–37.7; *P*, 0.026).

**Conclusions:**

The risk of rebleeding of a dural arteriovenous fistula with cortical venous drainage presenting with hemorrhage is increased in the first 2 weeks justifying early treatment. However, the magnitude of this increase may be considerably lower than previously thought. Treatment within 5 days was associated with a low rate of rebleeding and appears an appropriate timeframe.

Cranial dural arteriovenous fistulas (dAVFs) are abnormal anastomoses within the dura mater. They can be classified based on their venous drainage into those with or without cortical venous drainage (CVD). dAVF without CVD rarely causes intracranial bleeding, while those with CVD may cause hemorrhage.^1–3^

The risk of bleeding of dAVF with CVD has been studied in 5 cohort studies.^4–8^ Each study reported either overall annualized average hemorrhage rates or compared groups using Kaplan-Meier analyses. None have considered whether the rate of hemorrhage changes over time following presentation.

Several studies have reported rates of rebleeding following hemorrhagic presentation. One focused on early rebleeding reported that 35% of dAVF with CVD presenting with hemorrhage suffered a rebleed within the first 2 weeks.^9^ Five other studies have reported annualized rebleed rates varying from 7.4%^5^ to 46%,^10^ although none have reported if this risk changes over time.

The rate of early rebleeding has important clinical ramifications for when to treat and whether to treat. A very high rate of rebleeding may mandate immediate treatment, potentially with less experienced teams. Lower rebleed rates would support early but planned treatment and more subspecialization in the treatment of rare lesions (crude detection rate 0.16/100 000 adults per year).^11^ If there were no additional early rebleed risk at all, with annualized risks quoted at 7.4% per annum, some unstable patients may be more appropriate for delayed treatment and some very frail or elderly patients no treatment at all.

We therefore aimed to study how the rate of hemorrhage changes over time following a hemorrhagic presentation of cranial dAVF with CVD using the CONDOR (Consortium for Dural Fistula Outcomes Research) database.

## Methods

### Data Availability

The data that support the findings of this study can be requested from the CONDOR registry central repository (Dr Zipfel, zipfelg@wustl.edu); data requests are subject to approval of the CONDOR consortium.

### Patients

CONDOR comprises 14 centers in the United States, the United Kingdom, the Netherlands and Japan who have pooled their data from 1077 dAVF patients seen between 1990 and 2017. The contributing date ranges varied between centers. The formation and methodology of the CONDOR database has been described in detail elsewhere.^12^ All centers had institutional or ethics committee approval with waiver of written informed consent. Data of patients with dAVF and CVD were extracted. The TRIPOD guideline for reporting of prediction model development and validation was used and a checklist is presented in Table S1.

### Natural History Periods

The natural history period was defined as the time period from presentation with a hemorrhage related to the dAVF to the time of first treatment, rebleed, dAVF regression, or last follow-up. First treatment was considered as any attempt at treatment irrespective of whether it resulted in complete or partial cure of the dAVF. In cases of multiple hemorrhages during the natural history period, the number of hemorrhages was recorded, but patients were censored at time of first rebleed. The reasons some patients did not undergo treatment varied but included patient refusal poor clinical condition and comorbidities preventing treatment.

### Hemorrhage

All hemorrhages were confirmed radiologically on computed tomography (CT) or magnetic resonance imaging, or cerebrospinal fluid analysis from lumbar puncture. A hemorrhage was defined as dAVF related if it met any of the following criteria: (1) blood was located in the vicinity of the fistula’s venous outflow; (2) angiography showed contrast extravasation from the fistula; or (3) clinical notes indicated the dAVF as the likely cause of bleeding and no other cause more likely. This relationship was adjudicated by the investigators in local participating centers and all dAVF-related hemorrhages included if judged as certain or probable. The primary outcome was incidence of dAVF-related rehemorrhage on follow-up during the natural history period. Hemorrhages were further classified as intracerebral, subarachnoid, or intraventricular.

### Statistics

The incidence rate of further hemorrhage per person-time in the defined follow-up period was calculated, with 95% CIs using the Byar method and *P* by the Mid-P exact test, for both the first 2 weeks and the remainder of the subsequent year.

Baseline demographics and characteristics of the patients contributing to the early and late cohorts were compared with assess for confounding factors using χ^2^ and *t*-tests where appropriate. Incidence rates were compared by incidence rate ratio and difference with 95% CIs using the Byar method. There were no missing data, so no imputation was required.

## Results

Of 1077 patients with dAVF, 692 had CVD, while the remaining either had no CVD (n=373) or missing data (n=12). Of those with CVD, 37% (n=253) presented with a dAVF-related hemorrhage. There were a further 7 patients who suffered dAVF-related hemorrhage before treatment but after initial nonhemorrhagic presentation and had subsequent natural history period data; therefore, in total, 260 patients had CVD and hemorrhagic presentation. Ten patients were excluded for missing follow-up data. Overall, 250 patients formed the analysis cohort comprising 95 years of cumulative person-years natural history follow-up (Figure S1). Median time to censorship was 5 days (interquartile range 40 days); 242 patients were censored because of treatment, one because of spontaneous obliteration of their fistula and 7 patients experienced a further dAVF-related hemorrhage (Figure), approximating to an annualized rebleed rate of 7.3% (95% CI, 3.2–14.5). One patient suffered 2 dAVF-related rehemorrhages. There were no deaths following rebleeding, and all patients underwent subsequent treatment. Of those that rebled, fistula location was tentorial in 4, transverse sinus in 2, and convexity in 1. Of those that rebled, 43% were female, median age was 56 years (range, 0–85), and 57% had venous ectasia. Their presenting bleed was intracerebral in 4 cases, combined intracerebral and subarachnoid in 2 cases, and subarachnoid in 1 case.

**Figure.**
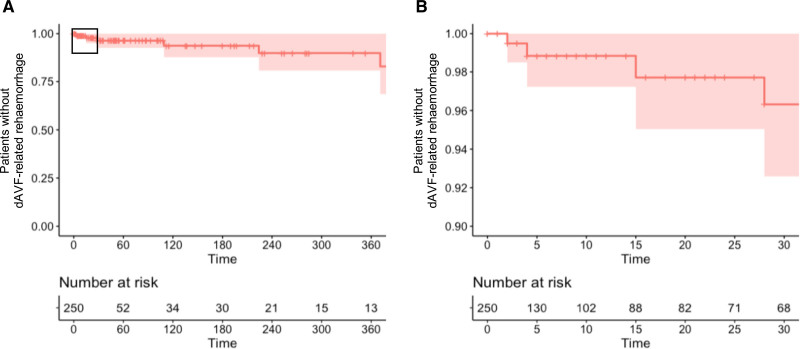
**Kaplan-Meier curve showing the survival of patients with cortical venous drainage (CVD). A**, All patients with CVD from time of first fistula related hemorrhage to rebleed for the first year of natural history follow-up and (**B**) for the first 30 d only (represented by boxed area in **A**). Patients were censored at first treatment or last follow-up if no treatment was received. Time in days. The red shaded areas represent the 95% CIs for patient survival without dural arteriovenous fistula (dAVF)-related rehemorrhage. Thirteen patients had follow-up beyond 1 y, which is not depicted.

The patients contributing to the early and late rebleeding risk are summarized in Table 1. The incidence rate in the first 2 weeks following a previous hemorrhage was 0.001106 per person-day of follow-up (95% CI, 0.0001854–0.003653), approximating to an early rebleed risk of 1.6% (95% CI, 0.3–5.1) in the first 2 weeks. After 2 weeks, the incidence rate of dAVF-related rehemorrhage was 0.000151 (95% CI, 0.0000487–0.000353) per person-day of follow-up until censorship, approximating to a late rebleed risk of 5.3% (CI, 1.7–12.4) for the remainder of the first year. The incidence rate ratio for rebleeding before and after 2 weeks was 7.3 (95% CI, 1.4–37.7; *P* 0.026) and the incidence rate difference 9.5 (95% CI, −5.8 to s24.9).

**Table 1.**
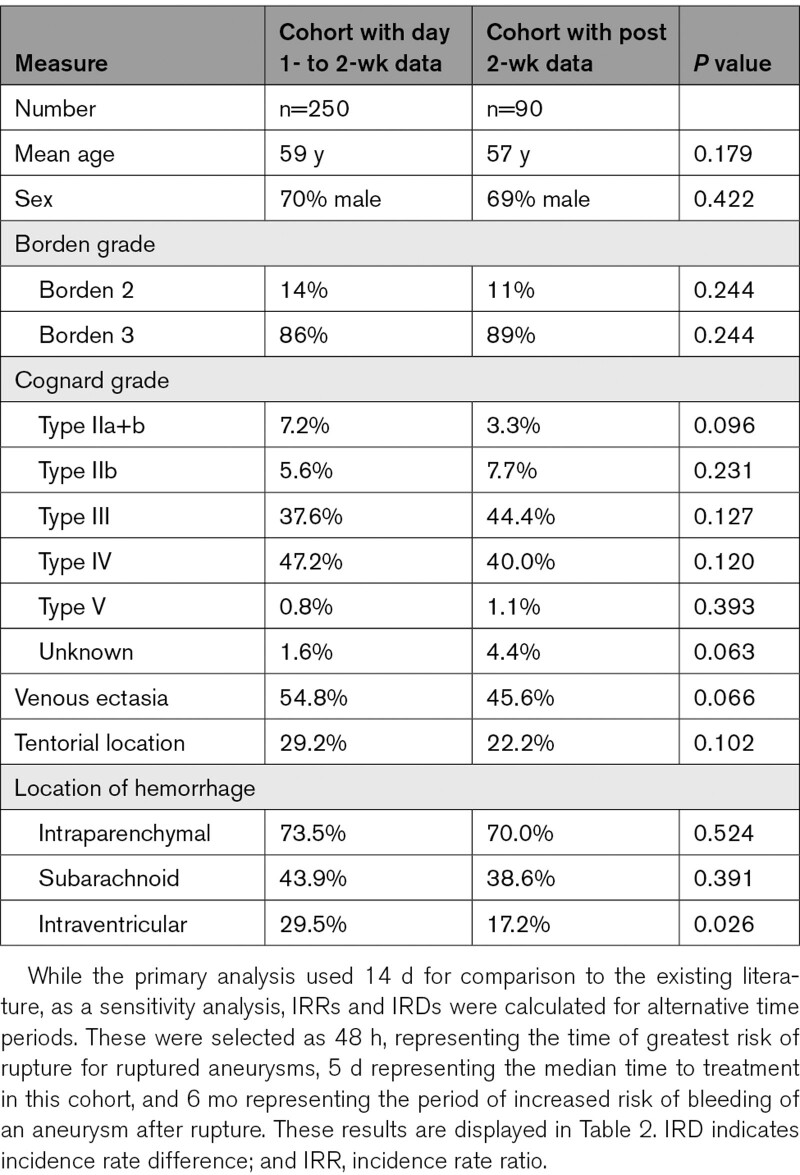
Characteristics of the Patients in the Early and Late Rebleeding Natural History Cohorts

**Table 2.**
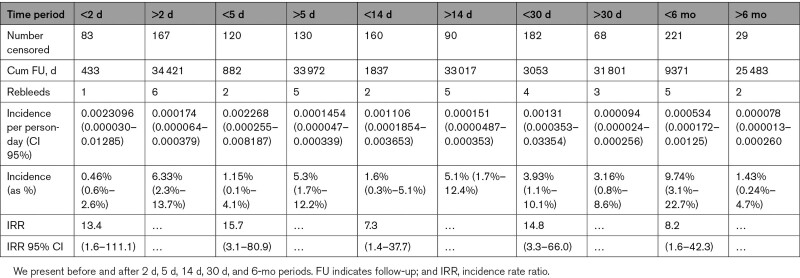
Secondary Analysis of Incidence Rates and Risk

## Discussion

Previous data suggested that the rebleeding risk in the first 2 weeks after hemorrhage was 35%.^9^ The current study challenges this estimate. Although we demonstrate for the first time that there is an early period of increased bleeding risk, its magnitude is significantly smaller than previously thought. Using the largest available database of dAVF with CVD, the incidence of bleeding was found to be 1.6% in the first 2 weeks, with an upper 95% confidence limit of 5.1%.

Rebleeding following dAVF hemorrhage is associated with a significant risk of morbidity or mortality.^4,13^ The long-term incidence of rebleeding in the current series is in agreement with similar published natural history cohorts.^5^ It therefore remains for the vast majority that early treatment is mandated. The lower than previously reported magnitude of the early risk raises questions, however, as to what constitutes early treatment. With a daily risk of 0.11% per day in the first 2 weeks, timing of treatment must carefully balance the best clinical outcome with an expeditious one. That balance of risk will vary depending on local pathways and resources. Transfer to more experienced institutions or time for further optimization before anesthesia may be warranted. In our series, a median time to treatment of 5 days was associated with a very low rate of rebleeding and would seem acceptable, although we would generally advocate the principle that earlier is better, up to the point where the quality of the procedure may be compromised.

The lower than previously reported early rebleed rate means that in a minority of patients who are very frail or medically unstable, the risk of rebleeding may be smaller than the risks of treatment. However, this is likely to be a very small group of patients in whom anesthetic risks are exceedingly high. It also raises the question whether patients presenting in coma should be treated acutely or upon showing signs of recovery. We have not presented any data on recovery from hemorrhage that would be needed to base any recommendations on. However, our in our experience by 5 days, imaging and clinical findings are sufficiently clear whether patients are likely to make a meaningful recovery or not to be able to decide whether to treat or not.

### Comparison to the Literature—Early Rebleeding

While risk of early rebleed was considerably smaller than previously reported, factors including sample size, selection bias, or different definitions of natural history and rebleeding may account for this.

The Duffau series was small (n=20), and both series were open to selection bias, primarily from loss of patients to treatment. This bias may be greater in the current series of patients, which were treated more recently. However, the most accepted predictor of repeat hemorrhage of dAVF with CVD is venous ectasia.^7^ In this series, 55% of cases and ≈40% in the Duffau series had venous ectasia, and this does not seem to explain the discrepancy in rebleed risk. All patients in the Duffau series were Cognard type III or IV fistula and while event rates did not permit subgroup analysis, ≈85% of our cohort was type III or IV of which 4 rebled (of 7). The only other putative predictor of rebleeding is tentorial location^14^ but was similar in both series.

The definition of rebleeding utilized in the present study is also different. In the prior study, only 4 of 20 cases (20%) had CT confirmed rebleeding, in others, it included the presence of differing aged blood at surgery and increased mass effect on serial angiographic imaging. In our study, all rebleeding was confirmed on imaging.

Not all dAVF in the Duffau series had an angiographic diagnosis. Notably, one case of recurrent acute subdural hemorrhage after previous subdural evacuation was attributed to dAVF rebleeding with no other objective evidence. All cases in our dataset had an angiographic diagnosis.

### Comparison to the Literature—Longer Term Rebleed

The overall annualized rate of rebleeding was 7.3% which is in agreement with published rates. Soderman et al^5^ reported an annualized rate of 7.4% in one of the few comparable datasets not included in the CONDOR. Other reports are generally limited by their small sample size and short follow-up, which are prone to overestimating annualized rebleeding risk. For example, a recent study across 3 cohorts with 169 dAVFs with CVD reported an annualized rebleed rate of 46%; however, mean follow-up was 0.3 years.^8^ Some studies included partially treated lesions and nonhemorrhagic presentations.^4^ Studies of patients specifically presenting with hemorrhage have not reported rebleeding incidence rates.^14,15^

### Strengths and Limitations

Both a strength and limitation of this study is its sample size. It is the largest analysis of patients with dAVF hemorrhage, and the first specifically reporting both early and late rebleeding rates. Despite this, the number of rebleeding events is low, limiting the degree of applicable analysis. However, given the known natural history, it is unlikely further significant amounts of natural history data will be collected in the future, and while some published series are not included in this analysis, these groups declined participation in the consortium. We therefore think this represents the best dataset likely to become available to address this issue. Other strengths are it is a multicenter study with well-defined methodology, and definitions developed by a consortium of experts and subject to careful quality control.

The main limitations are its retrospective nature and risk of selection bias. Ultra-early rebleeding either before admission to hospital, or before a scan is obtained may be undetected and lead to underestimation of the absolute early rebleed risk as has been observed in other conditions.^16^ In addition, the study included only tertiary referral centers and will not have included some patients admitted to secondary care and not referred either because they were too unwell to proceed with angiography and treatment, or due to an early rebleed. This may have led to underestimates of absolute rebleed rates, although in terms of generalizability to patients admitted to neurosciences centers, the presented data would be representative. Within this population, the median time to treatment was 5 days, and approximately one-third were treated within 2 days and thus early censorship is a limitation. In view that only 29 patients were available beyond 6 months means the late risks are particularly vulnerable to error. Clinicians could be selecting higher risk patients for earlier treatment. This could have led to an underestimate of the rebleeding risk.

## Conclusions

We demonstrate that the risk of rebleeding of a dAVF with CVD presenting with hemorrhage is significantly increased in the first 2 weeks. However, the magnitude of this increase appears lower than previously thought. The absolute incidence of a second bleeding episode in the first 2 weeks of 1.6%, and the long-term annualized risk of 7.3% means that treatment remains mandated in the vast majority of cases. In this series treatment within 5 days resulted in a low rate of rebleeding and appears to be an appropriate timeframe.

## Article Information

### Sources of Funding

Robert M. Starke’s research is supported by the NREF, Joe Niekro Foundation, Brain Aneurysm Foundation, Bee Foundation, and by the NIH (R01NS111119-01A1) and (UL1TR002736, KL2TR002737) through the Miami Clinical and Translational Science Institute, from the National Center for Advancing Translational Sciences and the National Institute on Minority Health and Health Disparities. Its contents are solely the responsibility of the authors and do not necessarily represent the official views of the National Institutes of Health.

### Disclosures

Dr Brinjikji reports compensation from Johnson and Johnson for consultant services; compensation from Stryker Corporation for consultant services; compensation from Johnson & Johnson Medical Devices & Diagnostics Group—Latin America, L.L.C. for consultant services; compensation from MicroVention, Inc for consultant services; compensation from MIVI Neurovascular for data and safety monitoring services; compensation from Medtronic Vascular, Inc for consultant services; stock holdings in Marblehead Medical LLC; compensation from Stryker for consultant services; compensation from MicroVention, Inc, for consultant services; and compensation from Medtronic USA, Inc for consultant services. Dr Abecassis reports stock options in remedy robotics; compensation from in neuro co for consultant services; and compensation from remedy robotics for consultant services. Dr Polifka reports compensation from DePuy Synthes Spine for consultant services. Dr Derdeyn reports compensation from noNO for data and safety monitoring services; compensation from Penumbra, Inc for data and safety monitoring services; employment by University of Iowa; and stock options in Euphrates Vascular. Dr Samaniego reports compensation from Medtronic for consultant services and compensation from Rapid Medical for consultant services and is a proctor with microvention. Dr Alaraj reports compensation from Johnson and Johnson and Cerenovus for consultant services. Dr Du reports compensation from Grand Rounds for consultant services; compensation from Grand Rounds for consultant services; compensation from National Institutes of Health for other services; and employment by Brigham and Women’s Hospital. Dr Kansagra reports compensation from Microvention and Penumbra for consultant services. Dr Gross reports compensation from Microvention and Medtronic for consultant services. Dr Kim reports compensation from Microvention for consultant services and is Spi Surgical co-founder. Dr Levitt reports grants from Stryker, Volcano Philips, and Medtronic and equity interest in Proprio, Cerebrotech and Synchron. Dr Starke reports consulting and teaching agreements with Penumbra, Abbott, Medtronic, InNeuroCo and Cerenovus.

### Supplemental Material

Figure S1

Table S1

## Supplementary Material



## Appendix

List of CONDOR collaborators: Washington University in Saint Louis: Gregory J. Zipfel , MD; Akash P. Kansagra , MD; Ridhima Guniganti, MD; Jay F. Piccirillo, MD; Hari Raman, MD; Kim Lipsey; Mayo Clinic: Giuseppe Lanzino , MD; Enrico Giordan , MD; Waleed Brinjikji , MD; Roanna Vine, RN; Harry J. Cloft, MD; David F. Kallmes, MD; Bruce E. Pollock, MD; Michael J. Link, MD; University of Virginia: Jason Sheehan , MD, PhD; Ching-Jen Chen , MD; Mohana Rao Patibandla, MCh; Dale Ding, MD; Thomas Buell, MD; Gabriella Paisan, MD; University of Washington: Louis Kim , MD; Michael R. Levitt, MD; Isaac Josh Abecassis, MD; R. Michael Meyer IV, MD; Cory Kelly; Wessex Neurological Center: Diederik Bulters, MBChB; Andrew Durnford, MA MSc, FRCS; Jonathan Duffill, MBChB; Adam Ditchfield, MBBS; John Millar, MBBS; Jason Macdonald, MBBS; University of Florida: W. Christopher Fox , MD; Adam J. Polifka , MD; Dimitri Laurent, MD; Brian Hoh, MD; Jessica Smith, RN; Ashley Lockerman, RN; University of Pittsburgh: Bradley A. Gross , MD; L. Dade Lunsford, MD; Brian T. Jankowitz, MD; University of Iowa: Minako Hayakawa, MD, PhD; Colin P. Derdeyn , MD; Edgar A. Samaniego , MD; Santiago Ortega Gutierrez, MD, MS; David Hasan, MD; Jorge A. Roa, MD; James Rossen, MD; Waldo Guerrero, MD; Allen McGruder, University of Illinois, Chicago: Sepideh Amin-Hanjani , MD; Ali Alaraj , MD; Amanda Kwasnicki, MD; Fady T. Charbel, MD; Victor A. Aletich, MS, MD (posthumous); Linda Rose-Finnell, University of Groningen, University Medical Center Groningen: J. Marc C. van Dijk , MD, PhD; Adriaan R.E. Potgieser, MD, PhD; University of Miami: Robert M. Starke, MD, MSc; Eric C. Peterson, MD; Dileep R. Yavagal, MD; Samir Sur, MD; Stephanie Chen, MD; Tokushima University: Junichiro Satomi, MD, PhD; Yoshiteru Tada , MD, PhD; Yasuhisa Kanematsu, MD, PhD; Nobuaki Yamamoto, MD, PhD; Tomoya Kinouchi, MD, PhD; Masaaki Korai, MD, PhD; Izumi Yamaguchi, MD, PhD; Yuki Yamamoto, MD; University of California, San Francisco: Adib Abla , MD; Ethan Winkler, MD, PhD; Ryan Phelps, Michael Lawton, MD; Martin Rutkowski, MD; Brigham and Women’s Hospital: Rose Du , MD, PhD; Rosalind Lai , MD; M. Ali Aziz Sultan, MD; Nirav Patel, MD; Ka
